# Medialized versus Lateralized Center of Rotation in Reverse Total Shoulder Arthroplasty: A Systematic Review and Meta-Analysis

**DOI:** 10.3390/jcm10245868

**Published:** 2021-12-14

**Authors:** Alessandra Berton, Lawrence V. Gulotta, Umile Giuseppe Longo, Sergio De Salvatore, Ilaria Piergentili, Benedetta Bandini, Alberto Lalli, Joshua Mathew, Russell F. Warren, Vincenzo Denaro

**Affiliations:** 1 Department of Orthopaedic and Trauma Surgery, Campus Bio-Medico University, Via Alvaro del Portillo, 200, Trigoria, 00128 Rome, Italy; a.berton@unicampus.it (A.B.); s.desalvatore@unicampus.it (S.D.S.); i.piergentili@unicampus.it (I.P.); benedettabandini.000@gmail.com (B.B.); albertolalli30@gmail.com (A.L.); denaro@unicampus.it (V.D.); 2 Centro Integrato di Ricerca (CIR), Campus Bio-Medico University, Via Alvaro del Portillo, 21, 00128 Rome, Italy; 3 Shoulder and Elbow Division of the Sports Medicine Institute, Hospital for Special Surgery, 535 E 70th Street, New York, NY 10021, USA; gulottal@hss.edu (L.V.G.); mathewj@hss.edu (J.M.); warrenr@hss.edu (R.F.W.)

**Keywords:** reverse shoulder arthroplasty, center of rotation, medialized, lateralized, Grammont, outcomes, scapular notching

## Abstract

One of the original biomechanical principles of reverse total shoulder arthroplasty (RTSA) is medialization of the center of rotation (COR) relative to the native level of the glenoid. Several authors have proposed the lateralized center of rotation, which is characterized by a lateralized (L) glenoid and medialized (M) humeral component. The aim of this review is to compare the clinical and functional outcomes of COR in medialized (M-RTSA) and lateralized (L-RTSA) RTSA in patients with uniform indications and treatment through a meta-analysis. A PRISMA-guided literature search of PubMed, Medline, Embase, Scopus, Cochrane Central Register of Controlled Trials, Cochrane Database of Systematic Reviews and Cochrane Clinical Answers was conducted from April to May 2021. Twenty-four studies were included in the qualitative synthesis, and 19 studies were included in the meta-analysis. Treatment with RTSA resulted in positive post-operative outcomes and low complication rates for both groups. Statistically relevant differences between L-RTSA group and M-RTSA group were found in post-operative improvement in external rotation with arm-at-side (20.4° and 8.3°, respectively), scapular notching rates (6.6% and 47.7%) and post-operative infection rates (1% and 7.7%). Both lateralized and medialized designs were shown to improve the postoperative outcomes. Nevertheless, a lateralized COR resulted in greater post-operative external rotation.

## 1. Introduction

Rotator cuff disorders are the most common cause of disability related to the shoulder [1,2]. Full-thickness rotator cuff tears are present in approximately 25% of individuals over 60 years old and 50% of people older than 80 years [2,3].

Reverse total shoulder arthroplasty (RTSA) is the procedure of choice for treatment of glenohumeral joint disease among patients with severe rotator cuff deficiency [4,5].

Although the medialized center of rotation (M-RTSA) has been associated with significant improvement in pain and function [6], complications have been reported, including scapular notching, fixation failure, infection, instability, glenoid component loosening, nerve injury and acromial fracture [7,8,9,10]. To overcome these problems and achieve better soft tissue balancing of the deltoid and the remaining rotator cuff muscles, several authors have proposed design modifications that increase center of rotation (COR) lateralization [4,11,12,13,14,15,16,17].

The lateralized COR (L-RTSA) is characterized by a lateralized glenoid position and medialized humeral component. This design has been reported to provide reduced impingement, improved length-tension of the rotator cuff and an improved deltoid “wrapping” [18,19,20,21,22] effect for a potentially lower dislocation rate [15,23,24,25,26,27,28]. Moreover, lateralization may also be achieved by autogenous bone graft augmentation, harvesting it from the humeral head [12,26,29]

The present study is meant to provide an updated systematic review and meta-analysis of outcomes relate to a medialized or lateralized COR [5,8,11,21,24,30,31].

To our knowledge, evidence identifying the best location for the COR is lacking, and no recent systematic reviews comparing the two prosthesis designs in a population of patients with standardized indications for RTSA have been reported in the literature.

The aim of this review and meta-analysis is to compare outcome measures, the number of revisions, the number of complications, scapular notching, and the active range of motion (ROM) between M-RTSA and L-RTSA.

## 2. Materials and Methods

### 2.1. Study Selection

The research question was formulated using a PICOS-approach: Patient (P); Intervention (I); Comparison (C); Outcome (O) and Study design (S). The aim of this systematic review is to describe whether patients (P) that underwent RTSA (I) with a lateralized COR reported better clinical and functional results compared to a medialized COR (C). The outcomes (O) assessed were: ROM, American Shoulders and Elbow Surgeons (ASES) score, Simple Shoulder Test (SST), Oxford Shoulder Test (OST), Absolute Constant-Murley score, Visual Analog score (VAS) for Pain, scapular notching, complications, revisions and self-assessed satisfaction.

The following study designs were included (S): Randomized Controlled Trials (RCT) and Non-Randomized Controlled Trials (NRCT), Prognostic (PG), Prospective (PS), Retrospective (RS), Case-Series (CS), Case-Control (CC), and Cohort (C) studies.

### 2.2. Inclusion Criteria

Only articles published in English were screened. Peer-reviewed articles of each level of evidence according to Oxford classification were considered. Studies reporting patients undergoing a primary RTSA were included, and the studies were considered eligible if they focused on a medialized or lateralized COR, or on both. The indication for RTSA of the patients enrolled in the trials was limited to cuff tear arthropathy, irreparable cuff tear or cuff tear associated with osteoarthritis.

### 2.3. Exclusion Criteria

Technical notes, letters to editors, instructional courses, or studies, including procedures other than reverse shoulder arthroplasty, were excluded. Articles were discarded if the mean follow-up was less than 12 months. Studies that considered revision RTSA, shoulder hemiarthroplasty and arthroscopic shoulder procedures, and RTSA combined with concurrent tendon transfer, were not included. In addition, articles reporting outcomes of patients with rheumatoid arthritis, acute fracture, post-traumatic fracture sequelae, tumors or active infection were not considered. In vitro, animal, cadaver and biomechanical studies were excluded. Studies that did not specify either the prosthesis design or COR or that were missing data were excluded.

Finally, data regarding patients who underwent procedures such as BIO-RSA were not considered in the statistical analysis, due to lack of standardization of the latter procedure; thus, in articles where the L COR group comprised such procedures, only data from the M COR group were included in the meta-analysis.

### 2.4. Search

A systematic review was performed using the Preferred Reporting Items for Systematic Reviews and Meta-analyses (PRISMA) guidelines [32]. Medline, EMBASE, Scopus, CINAHL and CENTRAL bibliographic databases were searched using the following string: ((scapular notching) OR (notching) AND (reverse shoulder arthroplasty) OR (reverse total shoulder) AND (medialized center of rotation) AND (lateralized center of rotation) AND (cuff tear arthropathy) OR (rotator cuff tear) OR (rotator cuff tear arthropathy)). Keywords were used both isolated and combined. Additional studies were searched among reference lists of selected papers and systematic reviews.

The search was performed by two of the authors (B.B. and L.A) from April to May 2021, and articles from the inception of the database to May 2021 were searched.

### 2.5. Data Collection Process

Data extraction was performed by two independent reviewers (B.B. and L.A.), and differences were reconciled by mutual agreement. In case of disagreement on inclusion or exclusion of articles, a third reviewer (S.D.S.) was consulted. The same authors (B.B. and L.A.) performed the review and organization of the titles in order to limit the bias.

The reviewers used the following screening approach: title and abstract were reviewed first, then the full articles. The full text of papers not excluded was evaluated and eventually selected after a discussion between the reviewers. In case of disagreement, the third reviewer (S.D.S.) decided. The number of articles included or excluded were registered and reported in the PRISMA flowchart. Standards reported by Moher et al. were adhered to in designing the PRISMA chart [29].

### 2.6. Data Items

General study characteristics extracted were: primary author, year of publication, type of study, level of evidence (LOE), sample size, mean age, gender totals and number of shoulders treated (Table 1). Moreover, prosthesis design, surgical approach, surgical characteristics (glenosphere size, glenoid tilt and humeral neck shaft angle, all sorted by M and L CORs) and follow-up were considered (in case of multiple time points, only the last follow-up was reported) (Table 2).

Outcome measures extracted included: Absolute Constant-Murley score; ASES score; Oxford Shoulder Score (OSS); Simple Shoulder Test (SST); Visual Analog Score (VAS) for pain (Table 3) revisions; complications and self-assessed satisfaction (Table 4); scapular notching, reported following the classification described by Sirveaux et al. [30] (Table 5); and active ROMs (forward flexion, abduction, external rotation with arm-at-side, external rotation in abduction, and internal rotation) (Table 6). All measurements were divided between the M-RTSA and the L-RTSA groups. VAS for pain was assessed either by a 0–10 scale, with 10 representing maximum pain [31,33,34,35], or by a 0–15 scale, with 15 being no pain [30,36,37,38,39]. Internal rotation was evaluated either by assigning a score to the maximum point reached by the thumb [14,25,30,31,33,34,40,41,42] or directly reporting by the vertebral level reached [11,36]. Pre-operative and post-operative values, including mean and standard deviation, were reported when present.

### 2.7. Study Risk of Bias Assessment

Given the designs of the included studies, the Risk of Bias (RoB 2) tool for randomized trials and the Risk of Bias in Non-Randomized Studies of Interventions (ROBINS-I) tool by Cochrane were used to assess the quality of each study [43,44]. Selected articles were independently rated by each reviewer (B.B., A.L.) and verified by a third one (S.D.S.) in case of disagreement.

### 2.8. Statistical Analysis

Categorical data were summarized as frequencies with percentages. Continuous data were summarized as mean values and standard deviations (SD) or ranges (minimum and maximum values). The subgroup meta-analysis was performed using a fixed-effect model or random-effect model, as applicable, and the Der-Simonian and Laird method for the estimation of the between-study variance. To quantify the heterogeneity among the studies, the I^2^ statistic was applied, with 50% defined as the threshold for significant heterogeneity [45]. A *p*-value less than 0.05 was considered statistically significant. All statistical analyses were performed using R software version i368 3.6.1.

## 3. Results

### 3.1. Study Selection

**Figure 1:**
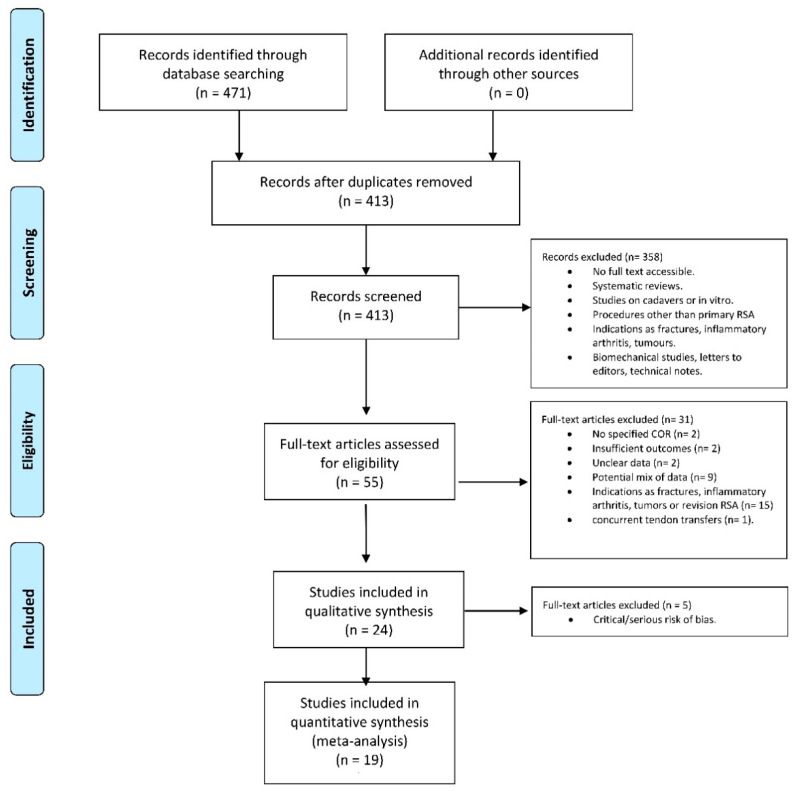
The PRISMA flow-chart of literature search.

The literature search identified 471 articles. No additional studies were found in the grey literature, and no unpublished studies were retrieved. Duplicate removal resulted in the exclusion of 58 studies, leaving 413 articles for screening, and 358 articles were excluded based on the title and abstract. Fifty-five articles were screened by full text, and 31 were excluded (insufficient outcomes, *n* = 2; unclear data, *n* = 2; no specified COR, *n* = 2; potential mix of data, *n* = 9; indications for RTSA as fractures, infection, inflammatory arthritis, tumors or revision arthroplasty *n* = 15; and patients with concurrent tendon transfers = 1). At the final screening, 24 articles met the selection criteria and were included in the review. The PRISMA flow-chart of literature search is reported in Figure 1.

### 3.2. Study Characteristics

The level of evidence (LOE) of each of the included was: two level I Randomized Control Trials [25,46], three level II Prognostic Studies [12,19,39,47], two level II Prospective Cohort Studies [18,42], two level III Retrospective Case-Control Studies [31,48], six level III Retrospective Cohort Studies [15,29,37,43,44,49] and nine level IV Retrospective Case Series Studies [30,35,37,38,39,42,47,50,51].

The 24 studies reviewed included a total of 2199 patients and 2276 shoulders, which were evaluated at a minimum follow-up of 12 months. The sample size for the M-RTSA group was 1138 patients, while the L-RTSA group comprised 1061 patients.

### 3.3. Quality of Evidence

The RoB 2 tool for RCTs and ROBINS-I tool for NRCTs were used to assess the methodological quality of each article [43,44]. Both the selected RCTs resulted in having a “low risk of bias” [25,46]. Out of the 22 NRCTs, nine were identified as “low risk of bias” studies [12,19,38,39,43,45,47,48,49]; eight were identified as “moderate risk of bias” studies [15,29,36,37,44,46,52,53]; four studies resulted in having a “serious risk of bias” [18,35,40,41]; and only one study was identified as a “critical risk of bias” study [39].

**Figure 2:**
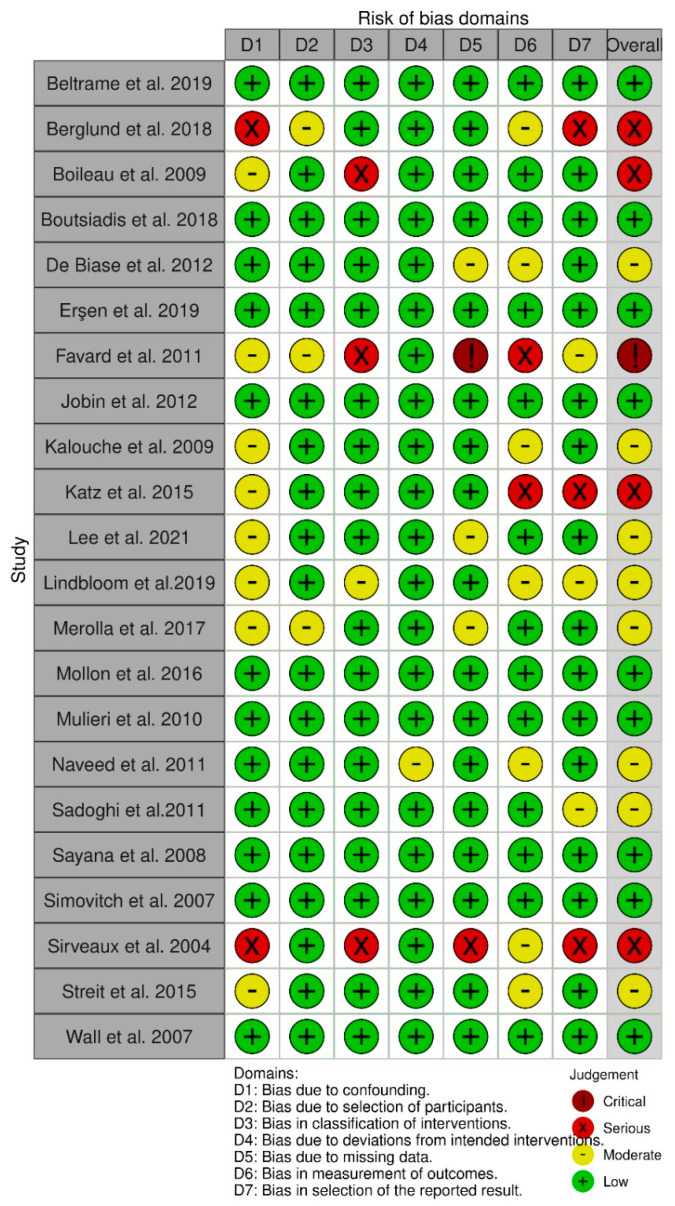
The risk of bias assessments for NRCTs studies.

**Figure 3:**
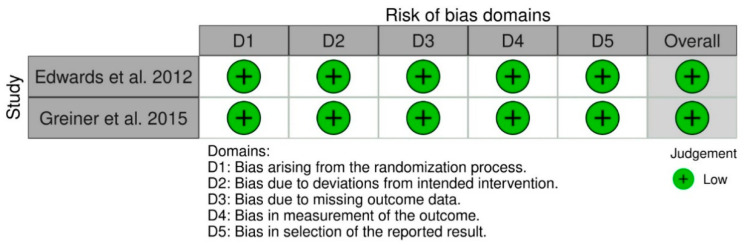
The risk of bias assessments for RCTs studies.

The risk of bias assessments for both RCTs and NRCTs are reported in Figure 2 and Figure 3.

### 3.4. Surgical Procedure

Twelve studies reported outcomes of M-RTSA [18,30,36,38,39,46,48,49,50,51,52,53], and four studies reported on the L-RTSA design [18,40,43,44]. Six studies compared standard M-RTSA to L-RTSA with or without BIO RSA [11,14,31,34,35,42], while two studies compared outcomes between standard M-RTSA and L-RTSA with bony-increased offset [25,33].

Patients in M-RTSA COR group received the following prosthesis designs:

Aequalis [12,19,26,36,41,42,51]; Aequalis II [33,34]; Delta [41,42,52]; Delta III [15,19,39,47,48,53]; Delta XTEND [53]; SMR [42,51]; and Zimmer [18].

Meanwhile, patients from the L-RTSA COR group were implanted with:

Aequalis [11,25]; Altivate [41]; Arrow [14,37]; Ascend Flex [12,29,45]; Encore [31]; Equinox [34,40]; RSP [35,47]; and RSP Monoblock [41].

In all the 24 studies selected, the chosen surgical approach was deltopectoral. Alternative surgical approaches were trans-acromial [35,42,48], superolateral [14,30], anterosuperior [38,39] and superior [37,50]. The choice of prosthesis design and surgical approach was based on the surgeon’s preference.

The glenosphere size reported ranged from 32 mm to 46 mm in diameter for both M and L COR groups, while the humeral neck shaft angle reported in the M group ranged from 150° to 155° and in the L group from 135° to 155°.

The complications reported were the following: bony spur, dislocation, infection, haematoma, glenoid loosening, neurologic complications, fractures, baseplate-glenoid fixation failure and luxation of glenoid (Table 4).

### 3.5. Meta-Analysis Results

As recommended by Sterne et al. [44], studies assessed as at serious or critical risk of bias were not included in the meta-analysis. Therefore, 19 studies [11,14,18,25,31,33,34,35,36,40,41,42,46,48,49,50,51,52,53] were included in the quantitative analysis.

#### 3.5.1. Outcome Measures

The data collected about outcome measures were Constant-Murley Score, ASES, SST, OSS and VAS scores. The Constant-Murley Score was collected from eight articles [12,15,26,43,46,49,51,52], the ASES score from five studies [19,43,44,51,53], the SST score from three studies [19,43,44] and the OSS score from two studies [48,50]. No articles on L-RTSA included in the meta-analysis assessed the OSS score; therefore, this questionnaire was not evaluated. No low and moderate risk of bias article measured the VAS; consequently, this score was not assessed. Constant-Murley Score, ASES and SST scores were included in the meta-analysis. No statistically significant differences between M and L in Constant-Murley Score, ASES and SST scores were found (*p* = 0.40, *p* = 0.96 and *p* = 0.76, respectively).

#### 3.5.2. Active ROMs

**Figure 4:**
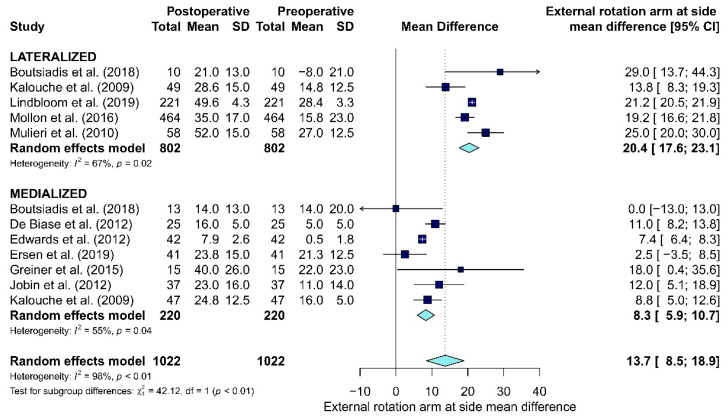
The forest plot of the mean difference for external rotation with arm-at-side between preoperative and postoperative follow-up in Lateralized and Medialized.

Data about active ROMs (i.e., active elevation or forward flexion, abduction, external rotation with arm-at-side, external rotation in abduction, and internal rotation) were collected (Table 6). The forward flexion was collected from 10 articles [12,15,19,26,38,43,44,46,49,51], abduction from seven studies [26,38,43,44,46,49,51], external rotation with arm-at-side from 10 articles [12,15,19,26,38,43,44,46,49,51], external rotation in abduction from three studies [15,19,26] and internal rotation from two articles [14,25]. No statistically significant differences between M and L were found in forward flexion (*p* = 0.93), abduction (*p* = 0.65) and external rotation in abduction (*p* = 0.06). Since no articles on L-RTSA included in the meta-analysis assessed the internal rotation, this ROM was not evaluated. A statistically significant difference was shown for external rotation with arm-at-side (*p* < 0.01). The mean difference for external rotation with arm-at-side between preoperative and postoperative follow-up in L was 20.4°, while the mean difference in M was 8.3° (Figure 4).

#### 3.5.3. Complications

**Figure 5:**
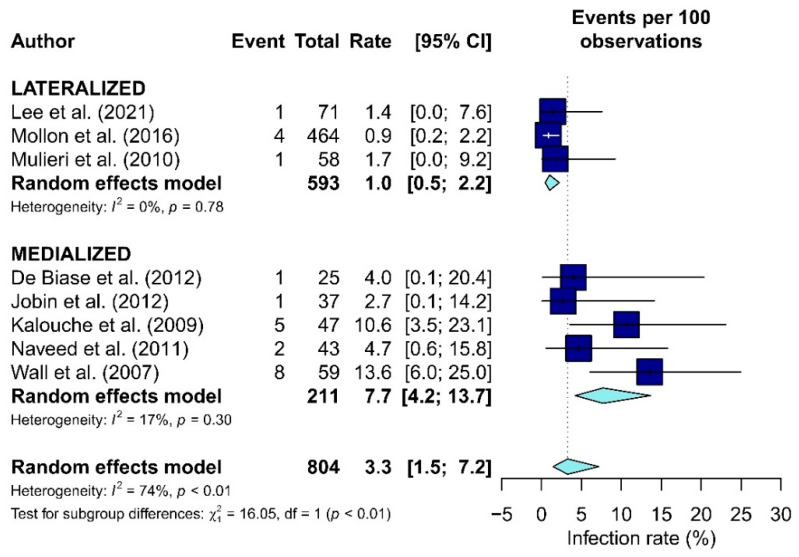
The forest plot of the infection rate in the Lateralized and Medialized.

Data about complications included in the meta-analysis were dislocation [14,33,34,35,36,46,51], infection [14,18,34,35,36,40,50,51], fracture [15,26,37,38,43,53], glenoid loosening [40,49] and revision [15,19,29,37,38,44,48,51,52,53] (Table 4). A statistically significant difference between L-RTSA and M-RTSA was found only for infections (*p* = 0.01). The infection rates in the L and M groups were 1% and 7.7%, respectively (Figure 5).

#### 3.5.4. Scapular Notching

**Figure 6:**
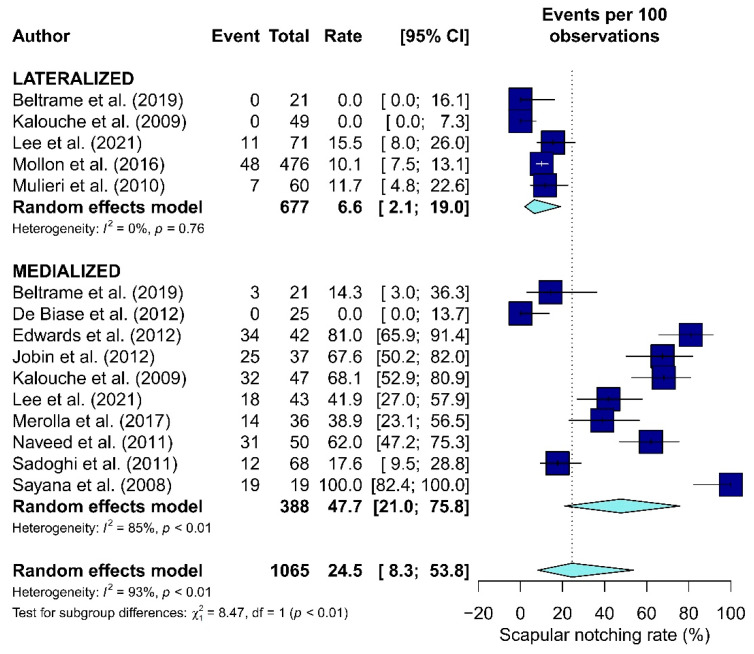
The forest plot of the scapular notching rate in the Lateralized and Medialized.

Twelve studies included data about scapular notching [15,19,29,37,38,43,45,46,48,51,52,53] (Table 5). The scapular notching rate was significantly higher in the M-RTSA group (47.7%) than the L-RTSA group (6.6%, *p* < 0.01, Figure 6).

## 4. Discussion

The use of RTSA for rotator cuff arthropathy has steadily increased over time due to improved medium-term outcomes [30,54]. The very first RTSA designs were fraught with glenoid implant failures. This problem was largely solved by Grammont’s design of a medialized COR since it converted shear forces across the glenoid implant–bone interface into compression forces. However, a medialized COR presented a separate set of issues such as scapular notching, limited rotation, instability and poor cosmesis of the shoulder. As glenoid fixation improved with the advent of porous ingrowth posts, central compression screws and locking fixation, RTSA designs began to lateralize the COR to address the issues seen with medialized COR implants. The purpose of this study was to determine which implant design—medial COR or lateral COR—provided better outcomes. We found that both implant designs predictably improve patient’s function and reduce their pain. However, our review shows that implants with a lateral COR may provide better rotation and less notching, with no increased incidence of implant failure.

Our findings are consistent with previous studies. Sirveaux et al. [30] reviewed 80 shoulders with a mean follow-up of 44 months. In this study, they reported pain relief in 96% of cases and a significant improvement of the Constant score. However, the study reported scapular notching in 63.6% of cases. Jobin et al. [18] reviewed 37 prostheses, at a mean follow-up of 16 ± 10 months, and noted good post-operative ASES scores. The main limitation of RTSA noted in the study was also scapular notching (reported in 68% of patients). L-RTSA was introduced to overcome this limitation, improving ROM and prosthesis stability [13]. Mollon et al. [40] retrospectively evaluated the results of RTSA with Equinox (Exactech, Inc., Gainesville, FL, USA), reporting a significant increase in abduction and internal rotation.

Several systematic reviews have previously compared L- and M-RTSA. Samitier et al. [5] has reported improvement in external rotation with the arm-at-side for L COR procedures using only the prostheses Reverse Shoulder Prosthesis (DJO Surgical, Austin, Texas), and Arrow Anatomical Shoulder System (Mulhouse, France) compared to the M COR ones in agreeance with our findings. However, their study did not include a meta-analysis and, in addition, the sample of patients in the current systematic review is greater and more homogeneous in terms of indication. The studies by Streit et al. [31] and Helmkamp et al. [23] also reported external rotation improvement in L-RTSA patients. Furthermore, a statistically significant difference was found in terms of scapular notching rates (*p* < 0.05), with a lower reported incidence for the L COR prostheses (Figure 6). These results are in line with the study by Alentorn-Geli et al. [55]. However, there was a discrepancy between the studies regarding the indication for RTSA: the current authors considered only cuff tear arthropathy, while Alentorn-Geli et al. did not apply any limitation in terms of inclusion criteria. Consistent with the results of the present systematic review, Heinkamp et al. also found scapular notching rates to be higher in the M group (L = 4.3%, M = 49%) [23].

No statistically relevant differences have been reported in the available literature on complications and revision rates [33,55]. The only statistically noteworthy divergence was found in infection rate, which was higher in the M-RTSA group (*p* < 0.01). This is difficult to explain and is most likely due to the fact that the first RTSA’s performed were with M-RTSA implants. Therefore, the indications were skewed to more difficult cases and the operative time was most likely longer given the learning curve of using RTSA. To our knowledge, no other systematic review reported statistically relevant differences in the infection rates between the two COR groups.

The strength of this review is the homogeneity of patients of the included studies. Only articles on patients who had undergone RTSA for rotator cuff arthropathy or an irreparable cuff tear associated or not with osteoarthritis were selected. Nevertheless, rotator cuff arthropathy is the most reliable indication for RTSA [36,56,57,58,59].

Additionally, data regarding BIO-RSA procedure were not included in the meta-analysis [12,26,29]: these techniques are not standardized and controlling for lateralization is difficult. These techniques can also be used to simply replace lost bone (i.e., B2 or B3 glenoid) without truly lateralizing the glenoid center of rotation.

Furthermore, all the considered articles presented a minimum follow-up of 12 months and revision surgeries were not considered. The studies were subjectively evaluated by the Cochrane risk of bias tools, RoB 2 and ROBINS-1 [43,44], in order to assess their potential risk of bias, and articles judged as having a serious or critical risk of bias were not included in the meta-analysis.

This study has some limitations. Firstly, the overall level of evidence of the studies included is low due to the limited presence of RCTs comparing M-RTSA and L-RTSA populations [26,51]. Moreover, the NRCTs included ranged from “low” to “critical” risk of bias according to ROBINS-I, thus only permitting the inclusion of low risk of bias and moderate risk of bias articles in the meta-analysis.

Furthermore, this systematic review does not consider the variability in global lateralization of implants [60]. Data regarding glenoid and humeral lateralization were combined into one group. For this reason, additional studies comparing the outcomes of humeral and glenoid lateralization are required, as recently proposed by Nabergoj et al. [61]. However, these functions differ in regard to force needed to generate overhead ROM as well restoration of ER, which is one of the main findings of the authors results. More control for implant type and lateralization would likely result in more meaningful data interpretation. Additionally, BIO RSA procedures were not included by the authors/in the review, nor was the role of the humeral neck shaft angle subject of evaluation.

The small sample size of some included articles downgraded the overall quality of the results. As observational studies constituted the main source for the analysis, selection bias and confounding due to diverse expectations in RTSA patients should be taken in consideration. Moreover, the statistically significant difference in reinfection between lateralized and medialized RSA should be influenced by the different duration (lateralized RSA (2011–2021) and medialized RSA (2007–2012)). In addition, the heterogeneous lengths of follow-up in the examined studies may contribute further inconsistencies. Furthermore, only English studies were included, limiting the available number of articles eligible for this review.

## 5. Conclusions

Both L and M designs have been shown to improve postoperative outcomes following RTSA. Nevertheless, it appears that the use of a lateralized COR is more likely to result in greater post-operative external rotation and lower rates of scapular notching. Further high-quality studies are required to compare L-RTSA with M-RTSA.

## Figures and Tables

**Table 1:** Primary author, year of publication, type of study, level of evidence (LOE), sample size, mean age, gender totals and number of shoulders treated of the included studies.

Author and Year	Type of Study	LOE	Sample Size TOT.	Sample Size M	Sample Size L	Shoulders	Mean Age ± SD (Range)	Mean Age M	Mean Age L	Gender TOT.		Gender M		Gender L	
										Males	Females	Males	Females	Males	Females
Beltrame et al., 2019	PS	IV	42	21	21	42		73 ± 8.2 (55–88)	77 ± 3.8 (68–85)	12	30	6	15	6	15
Berglund et al., 2018	RS; CS	IV	24	/	24	24						/	/		
Boileau et al., 2009	RS; CS	IV	40	40	/	42	70 (48–82)	70 (48–82)	/	7	33	7	33	/	/
Boutsiadis et al., 2018	PG	II	46	13 (I = 13)	10 (II = 10)	23	77 ± 7.5 (62–90)	77 ± 2 (I)	77 ± 2 (II)	9	37				
De Biase et al., 2012	RS; CS	IV	25	25	/	25	74.8 (69–87)	74.8 (69–87)	/	6	19	6	19	/	/
Edwards et al., 2012	RCT	I	42	42 (T = 20, Nt = 22)	/	42	69.0	71.8 ± 8.0 (T) 66.3 ± 9.8 (Nt)	/	19	23	10 (T) 9 (Nt)	10 (T) 13 (Nt)		
Erșen et al., 2019	RS; C	III	41	41	/	41	70.8 (57–84)	70.8 (57–84)	/	5	36	5	36	/	/
Favard et al., 2011	RS; CS	IV	489	464	/	509	76.1 (50–103)	76.1 (50–103)	/						
Greiner et al., 2015	RCT	I	15	15	/	15	75.4 (66–88)			7	8	7	8	/	/
Jobin et al., 2012	PS; C	II	37	37	/	37	76 (60–95)	76 (60–95)	/	10	27	10	27	/	/
Kalouche et al., 2009	RS; C	III	96	47	49	96		73.3 (58–88)	74.9 (52–89)	22	74	11	36	11	38
Katz et al., 2015	RS; CS	IV	134	/	134	140	72 (52–90)	/	72 (52–90)	34	100	/	/	34	100
Lee et al., 2021	RS; C	III	114	43	71	114		74.6 ± 4.9	73.7 ± 5.4	18	96	7	36	11	60
Lindbloom et al., 2019	RS; C	III	221	/	221	221		/		88	133	/	/	88	133
Merolla et al., 2017	RS; C	III	36	36	/	36		75.8 (55–88)	/	10	26	10	26	/	/
Mollon et al., 2016	RS; C	III	464	/	464	476	72.5 (53–90)	/	72.5 (53–90)	164	312	/	/	164	312
Mulieri et al., 2010	RS; CS	IV	58	/	58	60	71 (52–88)	/	71 (52–88)	16	42	/	/	16	42
Naveed et al., 2011	RS; CS	IV	43	43	/	50	81 (59–95)	81 (59–95)	/	7	36	7	36	/	/
Sadoghi et al., 2011	CC	III	66	66	/	68	66 (53–84)	66 (53–84)	/	30	36	30	36	/	/
Sayana et al., 2008	RS; CS	IV	18	18	/	19	72.8 (66–80)	72.8 (66–80)	/	6	12	6	12	/	/
Simovitch et al., 2007	PG	II	42	42	/	42	71.0 (54–85)	71.0	/	11	31	11	31	/	/
Sirveaux et al., 2004	RS; CS	IV	77	77	/	77	72.8 (60–86)	72.8	/	14	63	14	73	/	/
Streit et al., 2015	RS; CC	III	28 (10 CG)	9	9	28	70.6 + 74.7	70.9	70.4	5 + 6	13 + 4	3	6	2	7
Wall et al., 2007	PG	II	59	59	/	59			/					/	/

Abbreviations: PS = Prospective Study, RS = Retrospective Study, CS = Case Series Study, PG = Prognostic Study, RCT = Randomized Controlled Trial, C = Retrospective Cohort Study, CC = Case-Control Study, LOE = Level of Evidence, M = Medialized reverse total shoulder arthroplasty, L = Lateralized reverse total shoulder arthroplasty, SD = Standard Deviation, T = Glenoid tilt group, Nt = No glenoid tilt group, CG = Control Group, I = No glenoid lateralization group, II = No glenoid lateralization group, III = Glenoid lateralization group, IV = Glenoid lateralization group.

**Table 2:** Prosthesis design, surgical approach, surgical characteristics and follow-up of the included studies.

Author and Year	Surgical Approach	Prosthesis Design		Surgical Characteristics						Follow Up (Months)					
				Glenosphere Size (mm)		Glenoid Tilt		Humeral Neck Shaft Angle (°)		Mean		Range Max.		Range Min.	
		M	L	M	L	M	L	M	L	M	L	M	L	M	L
Beltrame et al., 2019	Deltopectoral	SMR	Ascend Flex					155	145	12	12				
Berglund et al., 2018	Deltopectoral	/	RSP	/	32, 32–4, 36, 36–4	/		/	135	/	43.4	/	77	/	24
Boileau et al., 2009	Deltopectoral (69%) Anterosuperior (31%)	Delta (81%) Aequalis (19%)	/	36 (95%) 42 (5%)	/		/		/	50	/	119	/	24	/
Boutsiadis et al., 2018	Deltopectoral	Aequalis (I)	Ascend Flex (II)	36 (40), 32 (6)	/	Inferior (10°)	/	155 (I)	145 (II)	39 ± 18	/	84	/	24	/
De Biase et al., 2012	Deltopectoral	SMR	/	36	/		/		/	27.5	/	46	/	24	/
Edwards et al., 2012	Deltopectoral	Aequalis	/	36	/	Inferior (10°), None	/	155	/	21	/		/	12	/
Erșen et al., 2019	Deltopectoral	Delta XTEND	/		/		/		/	34	/	67	/	12	
Favard et al., 2011	Anterosuperior (in 301) Deltopectoral (215) Transacromial (in 11)	Delta (in 461) Aequalis (in 66)	/		/		/		/	90	/		/	24	/
Greiner et al., 2015	Deltopectoral	Aequalis	Aequalis	36						22 ± 8.1	/	24	/	3	/
Jobin et al., 2012	Deltopectoral	Zimmer (27) Delta III (7) Aequalis (3)	/		/	Inferior (3° ± 12)	/		/	16 ± 10	/	26	/	6	/
Kalouche et al., 2009	Superolateral (M 44, L 41) Deltopectoral (M 3, L 8)	Delta III	Arrow	36						42.8	19.1	120	40	12	12
Katz et al., 2015	Superior (82.1%) Deltopectoral (17.8%)	/	Arrow	/	36 (83%)	/	Slightly Inferior	/	155	/	45	/	120	/	24
Lee et al., 2021	Deltopectoral	Aequalis II	Equinoxe	36	36			155	145			24		3	
Lindbloom 2019	Deltopectoral	/	RSP Mononblock AltiVate	/				/	135	/		/		/	
Merolla et al., 2017	Deltopectoral	Aequalis II		36, 42	/	Centered, Inferior	/	155	/	35.1	/	49	/	24	/
Mollon et al., 2016		/	Equinoxe		38 × 21 (256) 38 × 25 (10) 42 × 23 (189) 42 × 27 (11) 46 × 25 (10)	/	Not Inferior	/	145	/	38	/	93	/	22
Mulieri et al., 2010	Deltopectoral		RSP							/	52	/	101	/	24
Naveed et al., 2011	Deltopectoral Superior	Delta III	/	42 (men) 36 (women)	/		/		/	39	/	81	/	8	/
Sadoghi et al., 2011	Deltopectoral	Delta	/	36	/			150	/	42	/	96	/	24	/
Sayana et al., 2008	Transacromial Deltopectoral	Delta III	/		/		/		/	30	/	66	/	18	/
Simovitch et al., 2007	Deltopectoral	Delta III	/	36	/		/		/	43		96		24	
Sirveaux et al., 2004	Superolateral (72%) Deltopectoral 19% Transacromial (3.7%) Mixed (3.7%)		/	42 (3.7%)	/		/		/	44.5	/	24	/	97	/
Streit et al., 2015	Deltopectoral	Aequalis	Encore	36	32/−4 to 36			155	135	9.6	6.6				
Wall et al., 2007	Deltopectoral (98.7%)	Delta III, Aequalis	/							40		86		24 (81.6%)	

Abbreviations: I = No glenoid lateralization group, II = No glenoid lateralization group, III = Glenoid lateralization group, IV = Glenoid lateralization group, M = Medialized reverse total shoulder arthroplasty, L = Lateralized reverse total shoulder arthroplasty.

**Table 3:** Outcome measures of the included studies (Absolute Constant-Murley score, ASES score, Oxford Shoulder Score (OSS), Simple Shoulder Test (SST) and Visual Analog Score (VAS) for pain).

Author and Year		Constant-Murley Score				Absolute Ases Score				Simple Shoulder Test				Oxford Shoulder Score				Visual Analog Scale For Pain			
		L		M		L		M		L		M		L		M		L		M	
		Pre	Post	Pre	Post	Pre	Post	Pre	Post	Pre	Post	Pre	Post	Pre	Post	Pre	Post	Pre	Post	Pre	Post
Beltrame et al., 2019		39	71	41	70																
Berglund et al., 2018																					
Boileau et al., 2009		/	/	25.4 (7–59)	55.8 (0–11)	/	/			/	/			/	/			/	/	3.3 (0–11)	11.1 (3–15)
Boutsiadis et al., 2018		II = 21 ± 2.5 (8–30)	67 ± 4 (41–86)	I = 23 ± 3 (12–46)	62 ± 3 (45–71)		79 ± 5 (53–100)		75 ± 4 (53–98)		7 ± 1 (82–12)		7 ± 0.5 (4–11)								
																					
De Biase et al., 2012		/	/	30 (24–40)	64 (56–74)	/	/			/	/			/	/			/	/		
Edwards et al., 2012	T			13.1 ± 9.2	63.6 ± 12.3	/	/	56.3 ± 10.6	78.9 ± 10.8	/	/			/	/			/	/		
	Nt	/	/	15.7 ± 10.8	71.4 ± 14.9	/	/	59.6 ± 5.5	86.5 ± 11.6	/	/			/	/			/	/		
Erșen et al., 2019				38 ± 14	65 ± 11																
Favard et al., 2011		/	/	23.9 ± 9.9	61.5 ± 16.9	/	/			/	/			/	/			/	/	3.3 ± 3.3	12.2 ± 3.7
Greiner et al., 2015		/	/	26.1 ± 15.1	61.5 ± 16.0																
Jobin et al., 2012		/	/			/	/	24 ± 14	69 ± 24	/	/	2.0 ± 1.9	7.5 ± 2.9	/	/			/	/		
Kalouche et al., 2009		24.6 (11–40)	62.2 (49–75)	28.6 (14–45)	66.0 (50–86)																
Katz et al., 2015		26 (11–53)	64 (26–85)	/	/			/	/		8.66	/	/			/	/	3 (0–12)	13,7 (5–15)	/	/
Lee et al., 2021			69 ± 10.7	/	68.5 ± 10.2		79.0 ± 9.7		78.1 ± 10.2									/	1.6 ± 1.3		1.7 ± 1.5
Lindbloom 2019	M.					36 (33–40)	68 (64–72)	/	/	2 (1–2)	5 (5–6)	/	/								
	F					43 (38–47)	76 (71–81)	/	/	3 (2–3)	7 (6–7)	/	/								
Merolla et al., 2017		/	/	17.9	69.6													/	/	8.4	0.9
Mollon et al., 2016	Nn	35.0 ± 13.8	71.0 ± 14.2	/	/	38.2 ± 15.7	84.1 ± 17.1	/	/	3.5 ± 2.2	10.1 ± 2.6	/	/			/	/			/	/
	Y	32.7 ± 12.8	66.0 ± 13.9	/	/	34.3 ± 15.0	78.1 ± 21.8	/	/	3 ± 2.2	9.4 ± 3.0	/	/			/	/			/	/
Mulieri et al., 2010				/	/	33	75	/	/	1.6	6.5	/	/			/	/	6.3	1.9	/	/
Naveed et al., 2011		/	/	17	59	/	/	19 (14–23)	65 (48–82)	/	/			/	/	44 (40–51)	23 (18–28)	/	/		
Sadoghi et al., 2011		/	/	31.3 (14–63)	60 (19–88)	/	/			/	/			/	/	21.5 (12–41)	40.8 (32–50)	/	/		
Sayana et al., 2008		/	/	14.8	60.9																
Simovitch et al., 2007		/	/	38	78	/	/			/	/			/	/			/	/		
Sirveaux et al., 2004		/	/	22.60 (4–50)	65.5 (34–85)	/	/			/	/			/	/			/	/	2.7 (0–10)	13.4 (5–15)
Streit et al., 2015							71.0		75.1										0.7		0.3
Wall et al., 2007		/	/	22.8	59.7	/	/			/	/			/	/			/	/	3.5	12.3

Abbreviations: I = No glenoid lateralization group, II = No glenoid lateralization group, III = Glenoid lateralization group, IV = No glenoid lateralization group, ASES = American shoulder and elbow surgeon score, M = Medialized reverse total shoulder arthroplasty, L = Lateralized reverse total shoulder arthroplasty, Pre = Preoperative, Post = Postoperative, T = Tilt, Nt = No tilt, M. = Males, F = Females, Y = notching and Nn = no notching.

**Table 4:** Revisions, complications and self-assessed satisfaction of the included studies.

Author and Year		Self-Assessed Satisfaction		Complications		Revisions	
		L	M	L	M	L	M
Beltrame et al., 2019				None	None		
Berglund et al., 2018							
Boileau et al., 2009			37 benefited from the operation 34 very satisfied/satisfied 5 disappointed/dissatisfied		Bony spur (23)	/	1
Boutsiadis et al., 2018							
De Biase et al., 2012		/		/	Dislocation Infection	/	
Edwards et al., 2012	T	/		/	Dislocation (1)	/	1
	Nt	/		/		/	
Erșen et al., 2019							
Favard et al., 2011		/			Infection (27) Loosening (27) Dislocation (19) Haematoma (14) Neurologic (6)	/	13 +…
Greiner et al., 2015				/	Acromial fracture (2)		
Jobin et al., 2012		/		/	Infection (1) Baseplate-glenoid fixation failure (1)	/	2 (4%)
Kalouche et al., 2009				Disassembly (3) Dislocation (1)	Infection (5) Fracture (2)	7	4
Katz et al., 2015		91% better or much better 4% same 5% worse	/	Fracture (5), Nerve palsy (4), Humeral bearing failure (8), Infection (3), glenoid loosening (4)	/	12	/
Lee et al., 2021				Infection (1)	Acromial fracture (2), Dislocation (1)	0	0
Lindbloom 2019				Dissociation at Morse taper, Recurrent instability		2	
Merolla et al., 2017					Dislocation (2)		
Mollon et al., 2016	Nn		/	Fracture (8), Infection (4), Glenoid loosening (2)	/		/
	Y		/	Glenoid loosening (1), Fracture (3)	/		/
Mulieri et al., 2010		65% excellent 20% good 10% satisfactory 5% unsatisfactory	/		Fracture (4) Infection (1) Dislocation (1)	3	/
Naveed et al., 2011		/	16 patients no pain 15 mild pain 5 moderate pain 0 severe pain		Acromial erosion Fracture (2) Infection (2)	/	4
Sadoghi et al., 2011		/		/	Nerve lesion (1) Loosening of humeral stem (3) Luxation of glenoid (4)	/	7
Sayana et al., 2008				/	Glenoid loosening (1)	/	1
Simovitch et al., 2007			Subjective Shoulder value increased by 39% on average				
Sirveaux et al., 2004		/		/	Glenoid loosening (2) Infection (1)	/	3
Streit et al., 2015							
Wall et al., 2007		/	59.7% very satisfied; 33.3% satisfied; 5.9% uncertain; 1.1% disappointed	/	Dislcation (15) Infection (8) Glenoid fractures, humeral fractures, musculocutaneous nerve palsy, radial nerve palsy, glenoid sphere loosening and glenoid base loosening (<5)		

Abbreviations: M = Medialized reverse total shoulder arthroplasty, L = Lateralized reverse total shoulder arthroplasty, T = Tilt. Nt = No tilt, Y = notching and Nn = no notching.

**Table 5:** Scapular notching of the included studies.

Author and Year	Medialized COR						Lateralized COR					
	Scapular Notching		Grades of Notching (% or N)				Scapular Notching		Grades of Notching (% or N)			
	N	%	Grade I	Grade II	Grade III	Grade IV	N	%	Grade I	Grade II	Grade III	Grade IV
Beltrame et al., 2019	3	24	3	0	0	0	0	0	0	0	0	0
Berglund et al., 2018	/	/	/	/	/	/						
Boileau 2009	31	74	8	13	5	5	/	/	/	/	/	/
Boutsiadis et al., 2018												
De Biase et al., 2012	0	0	0	0	0	0	/	/	/	/	/	/
Edwards et al., 2012	15	86	25%	40%	10%		/	/	/	/	/	/
Edwards et al., 2012	19	75	36%%	45%%	5%%		/	/	/	/	/	/
Erșen et al., 2019							/	/	/	/	/	/
Favard et al., 2011					50%		/	/	/	/	/	/
Greiner et al., 2015					0	0						
Jobin et al., 2012	25	68					/	/	/	/	/	/
Kalouche et al., 2009	32	68	11	11	9	1	0	0	0	0	0	0
Katz et al., 2015	/	/	/	/	/	/	41	29	20	18	3	0
Lee et al., 2021	18	41.8	13	5	0		11	15.5	11	0	0	
Lindbloom et al., 2019	/	/	/	/	/	/						
Merolla et al., 2017	14	39.0	11	1	0	0	/	/	/	/	/	/
Mollon et al., 2016	/	/	/	/	/	/	48	10.1		2.1		
Mulieri et al., 2010	/	/	/	/	/	/	7	12	11.50%	1.90%	0	0
Naveed et al., 2011	31	62	5	7	11	8	/	/	/	/	/	/
Sadoghi et al., 2011	12	32	23%	3%	6%	0	/	/	/	/	/	/
Sayana et al., 2009	19	100	5	8	2	4	/	/	/	/	/	/
Simovitch et al., 2007							/	/	/	/	/	/
Sirveaux et al., 2004	49	64	26	10	7	6	/	/	/	/	/	/
Streit et al., 2015												
Wall et al., 2007							/	/	/	/	/	/

**Table 6:** Active ROMs (forward flexion, abduction, external rotation with arm-at-side, external rotation in abduction, and internal rotation) of the included studies.

Author and Year		ROM																			
		Forward Flexion (°)				Abduction (°)				External Rotation Arm-at-Side (°)				External Rotation in Abduction (°)				Internal Rotation			
		L		M		L		M		L		M		L		M		L		M	
		Pre	Post	Pre	Post	Pre	Post	Pre	Post	Pre	Post	Pre	Post	Pre	Post	Pre	Post	Pre	Post	Pre	Post
Beltrame et al., 2019			153 (120–180)		158 (120–180)		142 (100–170)		144 (100–180)		42 (30–60)		37 (20–40)						4.6		4.8
Berglund et al., 2018										−21	28										
Boileau et al., 2009		/	/	82 (20–180)	123 (40–170)	/	/			/	/	5 (−40–+70)	7 (−30–+60)	/	/			/	/		
Boutsiadis et al., 2018		II = 53 ± 22 (30–90)	149 ± 8 (90–175)	63 ± 21 (10–100)	148 ± 7 (100–170)		134 ± 9 (80–175)		134 ± 8.5 (90–170)	–8 ± 21 (−30–20)	31 ± 13 (15–60)	14 ± 20 (–30–50)	14 ± 13 (−10–35)					BUT (Lat. Thigh-Sacroiliac)	L3 (BUT-T12)	SI (BUT-T12)	L3 (BUT-T12)
																				Sacroiliac (BUT-T12)	L3 (BUT-T12)
De Biase et al., 2012		/	/	66 ± 9	148 ± 8	/	/	60 ± 9	115 ± 14	/	/	5 ± 5	16 ± 5	/	/			/	/		
Edwards et al., 2012	T	/	/	36.0 ± 45.6	148.0 ± 19.4	/	/	32.3. ± 37.4	141.8 ± 27.3	/	/	0.3 ± 1.3	7.4 ± 1.8	/	/			/	/		
	Nt	/	/	51.6 ± 49.1	156.6 ± 21.2	/	/	49.8 ± 49.0	155.9 ± 21.0	/	/	0.7 ± 1.8	8.3 ± 2.6	/	/			/	/	40.9	77.3
Erșen et al., 2019				77.5 (50–130)	111.6 (80–170)	/	/	84.5 (30–160)	108.8 (90–170)	/	/	21.3 (0–50)	23.8 (0–60)								
Favard et al., 2011		/	/	69.3 ± 34	128.6 ± 32.6	/	/			/	/	4.9 ± 17.6	10.6 ± 18.8	/	/	23.5 ± 23.3	42.1 ± 30.2	/	/		
Greiner et al., 2015		/	/	2.3 ± 2.5	6.8 ± 2.2	/	/	2.1 ± 2.1	6.7 ± 2.1	/	/	22 ± 23	Δ 18 ± 26	/	/	26 ± 31	Δ 33 ± 42	/	/	3.3 ± 2.8	5.3 ± 2.6
Jobin et al., 2012		/	/	38 ± 26	144 ± 19	/	/			/	/	11 ± 14	23 ± 16	/	/	18 ± 22	44 ± 30	/	/	L4 ± 3	L3 ± 3
Kalouche et al., 2009		61.5 (10.120)	134.7 (95–180)	70.3 (20–140)	140.8 (95–180)			/		14.8 (−20–70)	28.6 (0–60)	16.0 (−20–40)	24.8 (−10–60)	18.7 (−20–60)	51.5 (10–95)	25.6 (0–60)	48–0 (0–90)	4.5 (0–10)	6.4 (2–10)	5.2 (0–10)	6.0 (2–10)
Katz et al., 2015		73 (10–160)	132 (40–180)	/	/	61 (20–150)	108 (40–170)	/	/			/	/	30 (0–90)	54 (0–100)	/	/			/	/
Lee et al., 2021			132 ± 16		130 ± 16		125 ± 16		127 ± 14		48 ± 12		48 ± 14						L2 ± 2		L2 ± 4
Lindbloom 2019	M.	81 (72–90)	151 (142–159)			75 (68–82)	136 (126–146)			32 (24–39)	55 (46–64)							3 (3–4)	4 (4–5)		
	F	70 (63–78)	136 (128–144)			66 (59–73)	121 (113–130)			26 (19–33)	46 (38–54)							3 (2–3)	5 (4–5)		
Merolla et al., 2017		/	/	65	142					/	/	15	30					/	/	2.4	4.7
Mollon et al., 2016	Nn	89 ± 40	139 ± 26	/	/	72 ± 36	113 ± 27	/	/	16 ± 23	35 ± 17	/	/			/	/	3.3 ± 1.8	4.8 ± 1.6	/	/
	Y	89 ± 41	130 ± 30	/	/	70 ± 36	103 ± 23	/	/	14 ± 21	35 ± 16	/	/					3.3 ± 1.8	5.3 ± 1.5	/	/
Mulieri et al., 2010		53 (0–148)	134 (10–180)	/	/	49 (0–140)	125 (25–180)	/	/	27 (−20–70)	51 (−30–90)	/	/			/	/	S1	L2	/	/
Naveed et al., 2011		/	/	55	105	/	/		85	/	/			/	/			/	/		Buttock (35%) T12 (25%) Sacroiliac joint (20%) Waist (10%) Limited (10%).
Sadoghi et al., 2011		/	/	34	125	/	/	36	117	/	/	14.1	13.9	/	/			/	/		
Sayana et al., 2008		/	/			/	/			/	/			/	/			/	/		
Simovitch et al., 2007		/	/	65	115	/	/	63	111	/	/	16	20.5	/	/			/	/		
Sirveaux et al., 2004		/	/	73	138	/	/			/	/	3.50	11.20	/	/	17	40	/	/	4	4.8
Streit et al., 2015			115.6		143.9										35.0		28.3		−2.2		−1.8
Wall et al., 2007		/	/	76	142	/	/			/	/	5	7	/	/	29	43	/	/	L5	L3

Abbreviations: I = No glenoid lateralization group, II = No glenoid lateralization group, III = Glenoid lateralization group, IV = Glenoid lateralization group, T = Tilt, Nt = No tilt, ROM = range of motion, M = Medialized reverse total shoulder arthroplasty, L = Lateralized reverse total shoulder arthroplasty, Pre = Preoperative, Post = Postoperative, Y = notching, Nn = no notching, M. = males and F = females.

## Data Availability

The data presented in this study are available on request from the corresponding author.

## References

[1] Chakravarty K., Webley M. (1993). Shoulder joint movement and its relationship to disability in the elderly. J. Rheumatol..

[2] Tashjian R.Z. (2012). Epidemiology, natural history, and indications for treatment of rotator cuff tears. Clin. Sports Med..

[3] Berton A., Longo U.G., De Salvatore S., Sciotti G., Santamaria G., Piergentili I., De Marinis M.G., Denaro V. (2021). A Historical Analysis of Randomized Controlled Trials in the Management of Pain in Rotator Cuff Tears. J. Clin. Med..

[4] Berliner J.L., Regalado-Magdos A., Ma C.B., Feeley B.T. (2015). Biomechanics of reverse total shoulder arthroplasty. J. Shoulder Elb. Surg..

[5] Samitier G., Alentorn-Geli E., Torrens C., Wright T.W. (2015). Reverse shoulder arthroplasty. Part 1: Systematic review of clinical and functional outcomes. Int. J. Shoulder Surg..

[6] Petrillo S., Longo U.G., Papalia R., Denaro V. (2017). Reverse shoulder arthroplasty for massive irreparable rotator cuff tears and cuff tear arthropathy: A systematic review. Musculoskelet. Surg..

[7] Cho C.H., Jung J.W., Na S.S., Bae K.C., Lee K.J., Kim D.H. (2019). Is Acromial Fracture after Reverse Total Shoulder Arthroplasty a Negligible Complication?: A Systematic Review. Clin. Orthop. Surg..

[8] Youn S.M., Lee H.S., Rhee S.M., Rhee Y.G. (2021). Medialized vs. lateralized humeral implant in reverse total shoulder arthroplasty: The comparison of outcomes in pseudoparalysis with massive rotator cuff tear. J. Shoulder Elb. Surg..

[9] Kirzner N., Paul E., Moaveni A. (2018). Reverse shoulder arthroplasty vs BIO-RSA: Clinical and radiographic outcomes at short term follow-up. J. Orthop. Surg. Res..

[10] Shah S.S., Gaal B.T., Roche A.M., Namdari S., Grawe B.M., Lawler M., Dalton S., King J.J., Helmkamp J., Garrigues G.E. (2020). The modern reverse shoulder arthroplasty and an updated systematic review for each complication: Part I. JSES Int..

[11] Boutsiadis A., Lenoir H., Denard P.J., Panisset J.C., Brossard P., Delsol P., Guichard F., Barth J. (2018). The lateralization and distalization shoulder angles are important determinants of clinical outcomes in reverse shoulder arthroplasty. J. Shoulder Elb. Surg..

[12] Boileau P., Moineau G., Roussanne Y., O’Shea K. (2011). Bony increased-offset reversed shoulder arthroplasty: Minimizing scapular impingement while maximizing glenoid fixation. Clin. Orthop. Relat. Res..

[13] Frankle M., Siegal S., Pupello D., Saleem A., Mighell M., Vasey M. (2005). The Reverse Shoulder Prosthesis for glenohumeral arthritis associated with severe rotator cuff deficiency. A minimum two-year follow-up study of sixty patients. J. Bone Jt. Surg. Am..

[14] Kalouche I., Sevivas N., Wahegaonker A., Sauzieres P., Katz D., Valenti P. (2009). Reverse shoulder arthroplasty: Does reduced medialisation improve radiological and clinical results?. Acta Orthop. Belg..

[15] Valenti P., Sauzières P., Katz D., Kalouche I., Kilinc A.S. (2011). Do less medialized reverse shoulder prostheses increase motion and reduce notching?. Clin. Orthop. Relat. Res..

[16] Lädermann A., Denard P.J., Boileau P., Farron A., Deransart P., Terrier A., Ston J., Walch G. (2015). Effect of humeral stem design on humeral position and range of motion in reverse shoulder arthroplasty. Int. Orthop..

[17] Berton A., De Salvatore S., Candela V., Cortina G., Lo Presti D., Massaroni C., Petrillo S., Denaro V. (2021). Delayed Rehabilitation Protocol after Rotator Cuff Repair. Osteology.

[18] Jobin C.M., Brown G.D., Bahu M.J., Gardner T.R., Bigliani L.U., Levine W.N., Ahmad C.S. (2012). Reverse total shoulder arthroplasty for cuff tear arthropathy: The clinical effect of deltoid lengthening and center of rotation medialization. J. Shoulder Elb. Surg..

[19] Roche C.P., Diep P., Hamilton M., Crosby L.A., Flurin P.H., Wright T.W., Zuckerman J.D., Routman H.D. (2013). Impact of inferior glenoid tilt, humeral retroversion, bone grafting, and design parameters on muscle length and deltoid wrapping in reverse shoulder arthroplasty. Bull. Hosp. Jt. Dis..

[20] Cho N.S., Nam J.H., Hong S.J., Kim T.W., Lee M.G., Ahn J.T., Rhee Y.G. (2018). Radiologic Comparison of Humeral Position according to the Implant Designs Following Reverse Shoulder Arthroplasty: Analysis between Medial Glenoid/Medial Humerus, Lateral Glenoid/Medial Humerus, and Medial Glenoid/Lateral Humerus Designs. Clin. Shoulder Elb..

[21] Hamilton M.A., Roche C.P., Diep P., Flurin P.H., Routman H.D. (2013). Effect of prosthesis design on muscle length and moment arms in reverse total shoulder arthroplasty. Bull. Hosp. Jt. Dis..

[22] Routman H.D. (2013). The role of subscapularis repair in reverse total shoulder arthroplasty. Bull. Hosp. Jt. Dis..

[23] Helmkamp J.K., Bullock G.S., Amilo N.R., Guerrero E.M., Ledbetter L.S., Sell T.C., Garrigues G.E. (2018). The clinical and radiographic impact of center of rotation lateralization in reverse shoulder arthroplasty: A systematic review. J. Shoulder Elb. Surg..

[24] Costantini O., Choi D.S., Kontaxis A., Gulotta L.V. (2015). The effects of progressive lateralization of the joint center of rotation of reverse total shoulder implants. J. Shoulder Elb. Surg..

[25] Greiner S., Schmidt C., Herrmann S., Pauly S., Perka C. (2015). Clinical performance of lateralized versus non-lateralized reverse shoulder arthroplasty: A prospective randomized study. J. Shoulder Elb. Surg..

[26] Gutiérrez S., Levy J.C., Lee W.E., Keller T.S., Maitland M.E. (2007). Center of rotation affects abduction range of motion of reverse shoulder arthroplasty. Clin. Orthop. Relat. Res..

[27] Henninger H.B., Barg A., Anderson A.E., Bachus K.N., Burks R.T., Tashjian R.Z. (2012). Effect of lateral offset center of rotation in reverse total shoulder arthroplasty: A biomechanical study. J. Shoulder Elb. Surg..

[28] Lädermann A., Walch G., Lubbeke A., Drake G.N., Melis B., Bacle G., Collin P., Edwards T.B., Sirveaux F. (2012). Influence of arm lengthening in reverse shoulder arthroplasty. J. Shoulder Elb. Surg..

[29] Moher D., Liberati A., Tetzlaff J., Altman D.G., Group P. (2009). Preferred reporting items for systematic reviews and meta-analyses: The PRISMA statement. PLoS Med..

[30] Sirveaux F., Favard L., Oudet D., Huquet D., Walch G., Molé D. (2004). Grammont inverted total shoulder arthroplasty in the treatment of glenohumeral osteoarthritis with massive rupture of the cuff. Results of a multicentre study of 80 shoulders. J. Bone Jt. Surg Br..

[31] Streit J.J., Shishani Y., Gobezie R. (2015). Medialized Versus Lateralized Center of Rotation in Reverse Shoulder Arthroplasty. Orthopedics.

[32] Page M.J., McKenzie J.E., Bossuyt P.M., Boutron I., Hoffmann T.C., Mulrow C.D., Shamseer L., Tetzlaff J.M., Akl E.A., Brennan S.E. (2021). The PRISMA 2020 statement: An updated guideline for reporting systematic reviews. BMJ.

[33] Merolla G., Walch G., Ascione F., Paladini P., Fabbri E., Padolino A., Porcellini G. (2018). Grammont humeral design versus onlay curved-stem reverse shoulder arthroplasty: Comparison of clinical and radiographic outcomes with minimum 2-year follow-up. J. Shoulder Elb. Surg..

[34] Lee J.H., Chun Y.M., Kim D.S., Lee D.H., Shin S.J. (2021). Early restoration of shoulder function in patients with the Grammont prosthesis compared to lateralized humeral design in reverse shoulder arthroplasty. J. Shoulder Elb. Surg..

[35] Mulieri P., Dunning P., Klein S., Pupello D., Frankle M. (2010). Reverse shoulder arthroplasty for the treatment of irreparable rotator cuff tear without glenohumeral arthritis. J. Bone Jt. Surg. Am..

[36] Wall B., Nové-Josserand L., O’Connor D.P., Edwards T.B., Walch G. (2007). Reverse total shoulder arthroplasty: A review of results according to etiology. J. Bone Jt. Surg. Am..

[37] Katz D., Valenti P., Kany J., Elkholti K., Werthel J.D. (2016). Does lateralisation of the centre of rotation in reverse shoulder arthroplasty avoid scapular notching? Clinical and radiological review of one hundred and forty cases with forty five months of follow-up. Int. Orthop..

[38] Boileau P., Gonzalez J.F., Chuinard C., Bicknell R., Walch G. (2009). Reverse total shoulder arthroplasty after failed rotator cuff surgery. J. Shoulder Elb. Surg..

[39] Favard L., Levigne C., Nerot C., Gerber C., De Wilde L., Mole D. (2011). Reverse prostheses in arthropathies with cuff tear: Are survivorship and function maintained over time?. Clin. Orthop. Relat. Res..

[40] Mollon B., Mahure S.A., Roche C.P., Zuckerman J.D. (2017). Impact of scapular notching on clinical outcomes after reverse total shoulder arthroplasty: An analysis of 476 shoulders. J. Shoulder Elb. Surg..

[41] Lindbloom B.J., Christmas K.N., Downes K., Simon P., McLendon P.B., Hess A.V., Mighell M.A., Frankle M.A. (2019). Is there a relationship between preoperative diagnosis and clinical outcomes in reverse shoulder arthroplasty? An experience in 699 shoulders. J. Shoulder Elb. Surg..

[42] Beltrame A., Di Benedetto P., Cicuto C., Cainero V., Chisoni R., Causero A. (2019). Onlay versus Inlay humeral steam in Reverse Shoulder Arthroplasty (RSA): Clinical and biomechanical study. Acta Biomed..

[43] Sterne J.A.C., Savović J., Page M.J., Elbers R.G., Blencowe N.S., Boutron I., Cates C.J., Cheng H.Y., Corbett M.S., Eldridge S.M. (2019). RoB 2: A revised tool for assessing risk of bias in randomised trials. BMJ.

[44] Sterne J.A., Hernán M.A., Reeves B.C., Savović J., Berkman N.D., Viswanathan M., Henry D., Altman D.G., Ansari M.T., Boutron I. (2016). ROBINS-I: A tool for assessing risk of bias in non-randomised studies of interventions. BMJ.

[45] Higgins J.P., Thompson S.G. (2002). Quantifying heterogeneity in a meta-analysis. Stat. Med..

[46] Edwards T.B., Trappey G.J., Riley C., O’Connor D.P., Elkousy H.A., Gartsman G.M. (2012). Inferior tilt of the glenoid component does not decrease scapular notching in reverse shoulder arthroplasty: Results of a prospective randomized study. J. Shoulder Elb. Surg..

[47] Berglund D.D., Rosas S., Triplet J.J., Kurowicki J., Horn B., Levy J.C. (2018). Restoration of External Rotation Following Reverse Shoulder Arthroplasty without Latissimus Dorsi Transfer. JB JS Open Access.

[48] Sadoghi P., Vavken P., Leithner A., Hochreiter J., Weber G., Pietschmann M.F., Müller P.E. (2011). Impact of previous rotator cuff repair on the outcome of reverse shoulder arthroplasty. J. Shoulder Elb. Surg..

[49] Sayana M.K., Kakarala G., Bandi S., Wynn-Jones C. (2009). Medium term results of reverse total shoulder replacement in patients with rotator cuff arthropathy. Ir. J. Med. Sci..

[50] Naveed M.A., Kitson J., Bunker T.D. (2011). The Delta III reverse shoulder replacement for cuff tear arthropathy: A single-centre study of 50 consecutive procedures. J. Bone Jt. Surg. Br..

[51] De Biase C.F., Delcogliano M., Borroni M., Castagna A. (2012). Reverse total shoulder arthroplasty: Radiological and clinical result using an eccentric glenosphere. Musculoskelet. Surg..

[52] Simovitch R.W., Helmy N., Zumstein M.A., Gerber C. (2007). Impact of fatty infiltration of the teres minor muscle on the outcome of reverse total shoulder arthroplasty. J. Bone Jt. Surg. Am..

[53] Erşen A., Birişik F., Bayram S., Şahinkaya T., Demirel M., Atalar A.C., Demirhan M. (2019). Isokinetic Evaluation of Shoulder Strength and Endurance after Reverse Shoulder Arthroplasty: A Comparative Study. Acta Orthop. Traumatol. Turc..

[54] Baulot E., Chabernaud D., Grammont P.M. (1995). Results of Grammont’s inverted prosthesis in omarthritis associated with major cuff destruction. Apropos of 16 cases. Acta Orthop. Belg..

[55] Alentorn-Geli E., Samitier G., Torrens C., Wright T.W. (2015). Reverse shoulder arthroplasty. Part 2: Systematic review of reoperations, revisions, problems, and complications. Int. J. Shoulder Surg..

[56] Boileau P., Watkinson D., Hatzidakis A.M., Hovorka I. (2006). Neer Award 2005: The Grammont reverse shoulder prosthesis: Results in cuff tear arthritis, fracture sequelae, and revision arthroplasty. J. Shoulder Elb. Surg..

[57] Longo U.G., Candela V., De Salvatore S., Piergentili I., Panattoni N., Casciani E., Faldetta A., Marchetti A., De Marinis M.G., Denaro V. (2021). Arthroscopic Rotator Cuff Repair Improves Sleep Disturbance and Quality of Life: A Prospective Study. Int. J. Environ. Res. Public Health.

[58] Panattoni N., Longo U.G., De Salvatore S., Castaneda N.S.C., Risi Ambrogioni L., Piredda M., De Marinis M.G., Denaro V. (2021). The influence of psychosocial factors on patient-reported outcome measures in rotator cuff tears pre- and post-surgery: A systematic review. Qual. Life Res..

[59] Longo U.G., Berton A., De Salvatore S., Piergentili I., Casciani E., Faldetta A., De Marinis M.G., Denaro V. (2021). Minimal Clinically Important Difference and Patient Acceptable Symptom State for the Pittsburgh Sleep Quality Index in Patients Who Underwent Rotator Cuff Tear Repair. Int. J. Environ. Res. Public Health.

[60] Werthel J.D., Walch G., Vegehan E., Deransart P., Sanchez-Sotelo J., Valenti P. (2019). Lateralization in reverse shoulder arthroplasty: A descriptive analysis of different implants in current practice. Int. Orthop..

[61] Nabergoj M., Onishi S., Lädermann A., Kalache H., Trebše R., Bothorel H., Collin P. (2021). Can Lateralization of Reverse Shoulder Arthroplasty Improve Active External Rotation in Patients with Preoperative Fatty Infiltration of the Infraspinatus and Teres Minor?. J. Clin Med..

